# Immune Response in Thyroid Cancer: Widening the Boundaries

**DOI:** 10.1155/2014/125450

**Published:** 2014-09-25

**Authors:** Laura Sterian Ward

**Affiliations:** Laboratory of Cancer Molecular Genetics, Faculty of Medical Sciences, University of Campinas (FCM-Unicamp), Rua Tessália Vieira de Camargo 126, Barão Geraldo, 13083-970 Campinas, SP, Brazil

## Abstract

The association between thyroid cancer and thyroid inflammation has been repeatedly reported and highly debated in the literature. In fact, both molecular and epidemiological data suggest that these diseases are closely related and this association reinforces that the immune system is important for thyroid cancer progression. Innate immunity is the first line of defensive response. Unlike innate immune responses, adaptive responses are highly specific to the particular antigen that induced them. Both branches of the immune system may interact in antitumor immune response. Major effector cells of the immune system that directly target thyroid cancer cells include dendritic cells, macrophages, polymorphonuclear leukocytes, mast cells, and lymphocytes. A mixture of immune cells may infiltrate thyroid cancer microenvironment and the balance of protumor and antitumor activity of these cells may be associated with prognosis. Herein, we describe some evidences that immune response may be important for thyroid cancer progression and may help us identify more aggressive tumors, sparing the vast majority of patients from costly unnecessary invasive procedures. The future trend in thyroid cancer is an individualized therapy.

## 1. Introduction

Immune responses against differentiated thyroid carcinomas (DTC) and associations between inflammation and DTC have long been reported [1–4] and evidenced by a mixture of immune cells frequently found within, inside, or even surrounding primary thyroid tumors [2, 5]. These observations support the concept that the immune system may interfere in DTC progression [3, 4, 6].

Pathologists have long recognized that some tumors are marked infiltrated by cells of both innate and adaptive arms of the immune system, thereby reflecting inflammatory conditions arising in nonneoplastic tissues. In addition, clinicians have long been realizing that local immune response [1, 6] and concurrent chronic lymphocytic thyroiditis [1, 7] would be associated with favorable/unfavorable prognostic profile of patients with DTC. All these bedside observations stimulated investigations to unveil how the immune response is established in thyroid cancer and what is its influence on the outcome of patients with DTC.

## 2. Principles of Thyroid Carcinogenesis

### 2.1. Epidemiology and Risk Factors

Thyroid cancer accounts for around 2% of all human cancers [8]. Most of these patients will evolve very well with current therapy; however, 10–30% of them will present recurrent disease and part of them will eventually stop responding to radioiodine treatment and metastasize, contributing to 1,850 deaths due to thyroid tumors estimated to occur in the USA during 2013 [9].

Ionizing radiation is the most studied and consolidated risk factor for DTC. The thyroid may be irradiated more than other tissues because of its position in the body and its ability to concentrate iodine [10]. Mahoney et al. [11] have reported that the incidence of thyroid cancer after the Chernobyl accident has augmented in all areas of the Republic of Belarus and among all age groups, although children have suffered a more pronounced increase. Other studies also reported that there was a peak of PTCs after Chernobyl accident, when many children were exposed to high radiation doses [10, 12]. In 2011, Schonfeld et al. [13] presented an overview of the use of radiation for medical purposes and its significance for thyroid cancer, especially among children, who seem to be more susceptible to the effects of ionizing radiation; however, the authors concluded that X-rays do not seem to alter DTC risk. Thyroid cancers are one of the most common second cancers after radiotherapy during childhood for Hodgkin lymphoma, and significant increased risks of thyroid cancer have been observed even more than 40 years after childhood radiotherapy [14]. Several studies have been associating thyroid cancer with exposure to ^131^I, which can cause several atypical molecular alterations in genes such as* RET*,* NTRK1*,* RAS*, and* BRAF* [15–17].

Dietary iodine intake is among the possible environmental influences on the incidence and prevalence of thyroid disease in general and thyroid cancer in particular [18]. Increasing PTC has also been related to a high iodine intake [19]. Cardis et al. indicated that iodine deficiency increases the risk of ^131^I-related thyroid cancer [20]. These authors suggested that stable iodine supplementation in iodine-deficient populations may reduce the subsequent risk of radiation-related thyroid cancer [20].

Thyroid diseases occur with a marked higher frequency in women than in men for reasons that are not completely elucidated. PTC usually manifests during the reproductive age (30–49 years old), in a ratio of three to five females to one male and has the highest incidence in premenopausal women [21, 22]. Although PTC is more frequent in females, male sex is associated with a higher rate of malignancy among thyroid nodules [23], and several studies have suggested that male patients with DTC have worse survival [21, 24, 25]. Studying a very large cohort (36,725 patients), Oyer et al. [26] concluded that males with PTC and FTC tend to be older and have more advanced-stage disease relative to their female counterparts; however, there was no difference in disease-specific survival for men and women when they were compared by disease stage.

### 2.2. Molecular Genetic Alterations in Thyroid Cancer

The MAPK pathway plays an important role in DTC, and three of its genes (*BRAF*,* RET/PTC*, and* RAS*) can suffer mutually exclusive genetic alterations which have been implicated in the pathogenesis of PTC [27].* BRAF* mutations are the most frequent genetic alteration, occurring in approximately 45% of PTC cases [16]. The most common* BRAF* mutation causes a substitution of valine for glutamic acid at position 600 of the protein (V600E), but there are also an A > G transversion at gene position 1801 (K601E), the fusion with the A-kinase anchor protein 9 (*AKAP9*) gene, and small in-frame insertions or deletions around codon 600 [28–30]. All of these mutations are able to hyperactivate BRAF protein causing tumor progression or inducing features of aggressiveness [31], ultimately leading to the diminished or nule expression of several thyroid-specific genes, radioiodine uptake, and pronounced hypothyroidism [32]. As for* RET/PTC* translocations, they are present in sporadic and radiation-associated PTC, mostly in the last ones [33–35].* RET* translocations have not been related to bad prognosis of PTC but are important as targets for selective inhibition [27]. Several groups studied compounds with inhibitory effects on RET kinase activity and impairment of cell growth [36–38].* RAS* mutations are not restricted to a specific thyroid tumor type, being present in follicular adenomas, FTC, follicular variant of PTC, and at a high frequency in poorly differentiated cancers and anaplastic thyroid carcinomas [39, 40]. RAS has been shown to activate both the MAPK and the PI3K/AKT pathways in epithelial cells [27]. Mutations in* PI3KCA* gene have been described in 12–23% of ATC cases, restricted, in general, to undifferentiated thyroid components [41]. Other groups have reported somatic mutations within* PI3KCA* and/or gene amplification in FA, FTC, PTC, and ATC [41–43].* PI3KCA* alterations induce the activation of PI3K cascade through the enhanced activity of AKT, leading to thyroid cancer progression and possibly dedifferentiation [32]. Another gene frequently mutated in aggressive cases is* TP53*. Mutations in this gene are commonly observed in ATCs but are rarely described in DTCs [44–46], making it possible to hypothesize that this gene plays a role in late steps of thyroid cancer progression. A mutated p53 gene results in a marked loss of differentiated phenotype in rat thyroid cell line (PCCl3), including inhibition of the expression of thyroid-specific transcription factors [45, 47]. The loss of specific thyroid features is the main step for dedifferentiation and formation of very aggressive tumors with bad prognosis.

## 3. Principles of Tumor Immunology

A close relationship between immune response and cancer was first proposed by Virchow in 1863. Coley demonstrated that bacterial products were able to help inoperable cancer patients. Indeed, the subsequent application of* Bacillus* Calmette-Guerin (BCG) and other immunostimulants showed benefits that led to approval of their use in some solid tumors such as bladder cancer [48]. Thereafter, the capacity of the immune response to interfere with tumor progression has been evidenced by clinical and epidemiological reports. Then, the success of the usage of Herceptin for breast cancer and Ipilimumab for metastatic melanoma has stimulated many scientists throughout the world to explore the field of tumor immunology.

Presence of intratumor or peritumor infiltration of lymphocytes is evidence that the immune system may respond to malignant transformation. Several previous studies have shown that the high-grade density of CD8^+^ T cells in cancer cell nests was correlated with prognosis [49], and the presence of tumor infiltrating lymphocytes (TILs) was able to predict a better survival as an independent prognostic factor in various types of cancers including malignant melanoma [50], ovarian cancer [51], breast cancer [52], oral squamous cell carcinoma [53], esophageal cancer [54], and colon cancer [55]. The understanding of how immune cells and tumor interact came from different theories, as explained below.

Tumor transplantation models provide experimental support for the finding that tumors could be repressed by the immune system. These findings strongly suggested the existence of tumor-associated antigens and formed the basis of immune surveillance theory, which was postulated by Burnet and Thomas [56, 57]. Although there is excellent evidence to support the belief that immune surveillance mechanisms prevent the outgrowth of tumor cells induced by horizontally transmitted, ubiquitous, potential oncogenic viruses, there is much less evidence for immune surveillance acting against chemically induced tumors in syngeneic mice [49, 58].

During the malignant transformation, tumor cells may express tumor-specific and/or tumor-associated antigens. Tumor-associated antigens are macromolecules more frequently found in tumors, whereas tumor-specific antigens are macromolecules uniquely expressed in tumors [59]. Even though this group of antigens may be used as target for immunotherapy, the most important application of tumor antigen is for diagnosis or even to monitor therapeutic efficacy [60].

**Figure 1:**
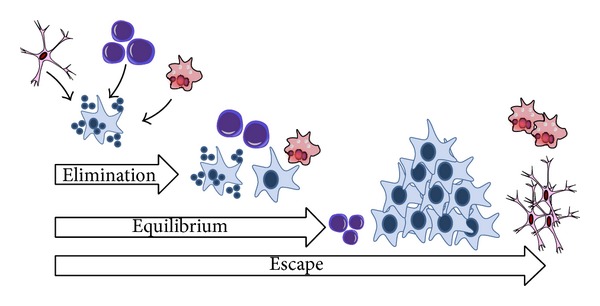
Three Es theory. This hypothesis suggests that the immune system and tumors may interact in three different stages. In the first stage, tumor cells are eliminated by the immune system. Elimination is followed by the second stage (equilibrium), where a selection process for less immunogenic tumor variants takes place until tumors are able to “escape” from immune surveillance (third stage).

In 2002, Dunn et al. proposed the cancer immunoediting hypothesis. According to this hypothesis, cancer immunoediting is responsible for both eliminating tumors and sculpting the immunogenic phenotypes of tumors that eventually form in immunocompetent hosts [61]. This hypothesis, called as “three Es theory,” suggests that the immune system and tumors may interact in three different stages (Figure 1). Tumor cells are initially eliminated by the immune system before becoming clinically detectable. This is equivalent to immunosurveillance. Elimination is then followed by an equilibrium phase, where a selection process for less immunogenic tumor variants takes place until tumors finally “escape” from immune surveillance [61]. Generally, by the time a tumor is clinically evident, it has escaped the host immune response and may even manipulate the immune system to promote tumor progression [62].

Clinical and experimental research has shown that tumor escape from immune recognition and tumor-mediated suppression of antitumor immunity can pose a significant obstacle to successful cancer therapy. Poschke et al. classified tumor escape from the immune system in two distinct mechanisms: camouflage and sabotage. Camouflage is active at the level of the tumor and can be ascribed to abnormalities in the expression of major histocompatibility complex (MHC) class I-restricted antigens, enabling tumors to take on a “stealth” phenotype, hiding from immune cell detection [63]. The other mechanism results from the tumor ability of progressing the host immune system to “sabotage.” Some tumors have the ability to manipulate parts of the body's own immune system to protect themselves against the host immune response. By doing that, tumors recruit or even induce immune cells like myeloid-derived suppressor cells (MDSCs) or regulatory T cells (Tregs). Under normal conditions, MDSCs and Treg serve as safeguards against overwhelming inflammation and lead to immune resolution [63]. The installation of these cells contributes to turning the permissive microenvironment for tumor progression.

One important mechanism of immune escape is T-lymphocyte dysfunction. T-lymphocytes are a common finding among TILs. The majority of T-cells are naive cells ignorant to the tumor, while a portion of T-cells show signs of activation but are functionally anergic or tolerant to the tumor [64]. Tumor-induced T-cell tolerance may be directed to self- and nonself-antigens [65–67]. Lee et al. studied anergic CD8+ T-lymphocytes. They identified circulating CD8+ T-cell populations specific for tumor-associated antigens in six of eleven patients with metastatic melanoma. These CD8+ T-lymphocytes presented two phenotypes: one, typical for memory/effector T-cells; the other, a previously undescribed phenotype expressing both naive and effector cell markers. Although these cells with latter phenotype have many of the hallmarks of effector T-cells, they were functionally unresponsive, unable to directly lyse melanoma target cells or produce cytokines in response to mitogens, suggesting that this population has been selectively rendered anergic* in vivo*. It should be highlighted that peptide stimulation of TAA-specific T-cell populations in other patients failed to induce substantial upregulation of CD69 expression, indicating that these cells may also have functional defects, leading to blunted activation responses [68].

Interestingly, Mortarini et al. observed that tumor-invaded lymph nodes of patients with melanoma were enriched with TILs. However, in tumor-invaded lymph nodes of most patients, CD8(+) T-cells directed to melanocyte differentiation antigens or to tumor-restricted antigens showed a precursor phenotype. Only in 7 of 23 cases antigen-specific CD8(+) T-cells in invaded lymph nodes showed a predominant preterminally differentiated phenotype. In the latter subset of patients, they detected staining for perforin and granzyme B in the cytoplasm of only a fraction of CD8^+^ cells present in neoplastic tissue. In these patients, they found perforin and granzyme B expressed on a higher fraction of CD8^+^ lymphocytes located in the residual lymph node tissue surrounding the invading tumor, with remarkably fewer positive lymphocytes among those intermingling with neoplastic tissue. In addition, analysis of invaded lymph node sections for morphological evidence of tumor necrosis and/or regression or apoptosis failed to show evidence for tumor destruction in all of the lesions, suggesting that incomplete CD8^+^ T-cell maturation and reduced expression of cytotoxic factors in CD8^+^ T-cells may impair tumor cell destruction in invaded lymph nodes [69].

## 4. Relationship between Chronic Lymphocytic Inflammation and DTC

Hashimoto's disease and Graves' disease are the two most common forms of autoimmune thyroiditis. Both are characterized by lymphocytic infiltrate and autoreactivity against thyroid autoantigens.

Chronic lymphocytic thyroiditis (CLT) is an autoimmune disease characterized by fibrosis, lymphocytic infiltration, and parenchymal atrophy of thyroid tissue. Hashimoto's thyroiditis (HT) is characterized by infiltration of the thyroid gland by immune cells, often followed by hypothyroidism due to destruction and eventual fibrous replacement of the parenchymal tissue. In HT, the immune system produces autoantibodies to thyroid-specific antigens, considering that thyroglobulin (Tg) and thyroperoxidase (TPO) are the two primary antigens in thyroid autoimmunity [70, 71]. Its unclear etiopathogenesis strongly indicates an autoimmune background, associated with T-helper lymphocyte (CD4+) activation by class II human leukocyte antigen system cells (MHC class II: HLA-DR3, HLA-DR4, and HLA-DR5). Conversely, cells recruit cytotoxic lymphocytes (CD8+), thus facilitating a release of cytokines that damage thyroid follicular cells and activating B lymphocytes [72].

HT is characterized by a gradual loss of thyroid function, goiter, and T-cell infiltration in histology, affecting women more often than men, with a sex ratio of 7 : 1, and occurring in genetically susceptible populations, but lacking a strong association with HLA. The overridden feature of HT is the progressive depletion of thyroid epithelial cells, which are gradually replaced by mononuclear cell infiltration and fibrosis [73, 74]. In thyroiditis, especially HT, parenchyma of thyroid gland is progressively lost and replaced by cells of the inflammatory infiltrate that produce chemokines, cytokines, and growth factors, most of which are under NF-*β* transcriptional control. The persistent stimulation of residual thyrocytes with such molecules could induce the activation of NF-*β* in follicular cells, thereby creating a functional network between thyroid epithelial cells and inflammatory cells [74].

A functional relationship between chronic inflammation and cancer was first proposed by Virchow, 1863, and has been sustained by clinical and epidemiological evidence [4, 75–79]. The relationship between CLT and PTC was first proposed by Dailey et al., 1955 [80]. Since this initial description, the association between the diseases has been repeatedly reported and highly debated in the literature, remaining controversial. However, it is important to distinguish two arising questions: (A) is chronic lymphocytic thyroiditis a risk for thyroid cancer development? (B) Would concurrent chronic lymphocytic thyroiditis be associated with favorable/unfavorable prognosis or patients with thyroid cancer?

### 4.1. Is Chronic Lymphocytic Thyroiditis a Risk for Thyroid Cancer Development?

The follicular epithelium in CLT is not homogenous throughout the entire organ and incidental multifocal papillary microcarcinomas are often identified. In addition, some reports from clinical observations suggest that atypical lesions that are not microcarcinomas should be classified as either reactive or premalignant “dysplastic” foci [81]. According to Chui et al., it is not unusual to observe distinct microscopic foci of follicular epithelial proliferations in areas of severe inflammation, which lack invasive growth, papillary architecture, or intranuclear pseudoinclusions, and thus do not qualify as papillary microcarcinoma. Chui et al. proposed designating these small atypical lesions as “follicular epithelial dysplasia.” Follicular epithelial dysplasia is morphologically distinct from the surrounding parenchyma and is often found in patients with severe CLT and multifocal papillary microcarcinoma [81]. They found that follicular epithelial dysplasia lesions were positive for TTF-1 and thyroglobulin, though some also expressed p63. Similar to PTC, strong diffuse staining was observed for HBME-1, cytokeratin 19, galectin-3, and cyclin-D1. In contrast, normal thyroid, reactive atypia, and follicular nodular disease were negative or, at most, exhibited focal weak staining for HBME-1, cytokeratin 19, and galectin-3. The results of this study showed the presence of atypical microscopic lesions in CLT with an immunohistochemical profile similar to PTC, suggesting the idea of a premalignant lesion preceding PTC, arising in the context of severe chronic inflammation [81]. What do epidemiological studies tell us?

It is not a novelty that CLT is a risk for several types of cancer. A number of studies have examined the possible association of CLT with subsequent types of cancer. In fact, CLT is associated with an increased risk for myeloproliferative and lymphoproliferative neoplasms [82], malignant lymphoma of the thyroid [82–84], and breast cancer [85].

In retrospective studies, the rates of malignancy in thyroid nodules in patients having HT ranged from 0.5% to 53%; in many [5, 86–90], but not all [56, 91], the rate of malignancy in thyroid nodules was considered to be higher if there was concomitant HT than if there was no HT. Anil et al. [92] performed a single-center prospective study. Prospective data were gathered on all patients newly diagnosed with thyroid nodules who were sent for fine-needle aspiration cytology (FNAC). If a patient had at least one positive thyroid autoantibody, then the patient was defined as having HT with thyroid nodules. There were 164 patients with thyroid nodules associated with HT (HT group). There were 551 patients with thyroid nodules without HT (control group). The malignancy rate of the nodules in HT group was 1.0% and that of the nonautoimmune control group was 2.7%, which was not significant. A possible limitation of this study was that only 4.9% of our HT group and 10% of our control group had thyroidectomy for their thyroid nodule(s) [92].

Boi et al. [90] assessed the association between thyroid autoimmunity and thyroid cancer in a retrospective series of unselected thyroid nodules submitted to FNAC to avoid the selection bias of surgical series. Ultrasound- (US-) guided FNACs were obtained from 590 patients with single thyroid nodules and positive (ATA+, *n* = 197) or negative (ATA−, *n* = 393) serum anti-thyroid antibody (ATA). A higher prevalence of indeterminate risk of malignancy (28.9% versus 21.4%, *P* < 0.05) and suspected malignancy (18.8% versus 9.2%, *P* < 0.001) and lower prevalence of low risk or benign cytology (52.3% versus 69.5%, *P* < 0.001) were found in ATA+ versus ATA− nodules, respectively. Multivariate logistic regression analysis evidenced that ATA+ conferred a significant risk of malignant cytology or suspicion of that, independently of age and sex. In 106 patients where thyroidectomy was carried out, thyroid cancer was found in 54/61 (88.5%) patients with malignant cytology nodules or suspicion of them, with similar positive predictive value for cancer in ATA+ (96.4%) and ATA− (81.8%) nodules [72].

Recently, Chen et al. [93] performed a nationwide cohort study. The Taiwanese national health insurance research database was used to identify 1521 newly diagnosed HT patients from 1998 to 2010 and 6084 frequency-matched non-HT patients. Hashimoto's thyroiditis patients were more likely to be diagnosed with thyroid and colorectal cancer, with an adjusted hazard-ratio of 11.8 and 4.76, respectively. Specifically, the HT cohort had a 49.4-fold risk of developing thyroid cancer during the first 3 years when compared with the non-HT cohort. According to the authors, the increased thyroid cancer risk appears in early years, since HT has been diagnosed due to the coincident findings of thyroid cancer. This may reflect the result of early thyroid cancer diagnosis from hard investigation by diagnosis and treatment of HT [93].

### 4.2. Would Concurrent Chronic Lymphocytic Thyroiditis Be Associated with Favorable/Unfavorable Prognosis or Patients with Thyroid Cancer?

It remains unclear whether the association with a CLT or even an autoimmune disorder could affect the prognosis of DTC. In fact, a worse prognosis was reported in some series [94, 95], whereas most studies showed either a protective effect of thyroid autoimmunity [6, 58, 96, 97] or a similar behavior between cancer with and without thyroiditis [3, 4].

At the end of a mean follow-up of 8.9 ± 2.2 years, in a retrospective study comparing 85 Chinese patients with both PTC and HT and 1,708 PTC patients without HT and 8 patients with follicular thyroid carcinoma (FTC) and HT and 201 FTC without HT, Kim et al. evidenced that patients presenting concurrent HT had less aggressive tumors and a better outcome [49]. In addition, in a retrospective analysis comparing 195 patients with PTC, Yoon et al. showed that patients presenting concurrent CLT had less aggressive tumors [98].

In our cohort, the presence of concurrent CLT was also significantly correlated with favorable prognostic features including female gender, no extrathyroidal tumor invasion, absence of metastasis at diagnosis, and small tumors, thus confirming the tight relationship between concurrent autoimmunity and histopathological features of low aggressiveness [99]. Using the same criteria introduced by Huang et al. to define recurrence, metastasis, and disease-free survival, a log-rank test also confirmed that the absence of CLT was more frequent in our patients who had recurrences, suggesting that autoimmune activity against the gland may exert a protective effect on the outcome of DTC patients [100].

Recently, Dvorkin et al. [101] underwent a retrospective study investigating 753 patients with DTC divided into two groups of patients with and without HT at diagnosis. HT was associated with smaller primary tumor and less lymph node involvement at presentation. When matched groups were compared, patients with HT received less additional radioactive iodine treatments and showed lower rates of persistence at 1 year and higher rates of disease remission at the end of follow-up. Regarding multivariate analysis, HT was predictive of a lower rate of lymph node involvement and persistent disease at the end of follow-up, suggesting that HT is associated with a less aggressive form of differentiated thyroid cancer and a better long-term outcome. Likewise, Lun et al. [102] performed a case-control study of 2478 patients who underwent thyroidectomy. Compared with patients with benign thyroid nodular disease, patients with PTC showed a significantly higher prevalence of HT. Patients with PTC and HT were younger, with female predominance, and had smaller sized tumors with less advanced TNM stage when compared with those without HT, indicating a better prognosis.

## 5. Molecular Link between Inflammation and DTC

Both a causal association and a noncausal association between thyroid cancer and CLT have been proposed. The molecular mechanism that links inflammation and cancer is not completely clear so far. A link between thyroid cancer, in particular the PTC histotype, and thyroid autoimmunity has long been recognized, although the precise relationship between the two diseases remains subject of debate. Herein, we list some evidences of molecular mechanisms that may be involved in both thyroid cancer and thyroid autoimmunity.

Pathways of immune activation could exert a role in thyroid cancer and CLT link. Toll-like receptor (TLR) comprises a family of cell surface receptors involved in the recognition of pathogen-associated signature molecules that signal the activation of innate and adaptive immunity [103–107]. TLR family consists of more than ten members, and TLR3 had been reported to be restricted primarily to dendritic cells of the immune system [107, 108]. Harii et al. showed that TLR3 can be functionally overexpressed in cultured human thyrocytes by stimuli. Immunohistochemistry showed that TLR3 protein is overexpressed in human thyrocytes surrounded by immune cells in all patients diagnosed with Hashimoto's thyroiditis, suggesting that TLR3 overexpression can induce an innate immune response in thyrocytes, which may be important in the pathogenesis of HT and in immune cell infiltrates [103]. McCall et al. showed that PTC cells basally express TLR3 RNA and that TLR3 signal systems are functional in these cells. High basal TLR3 levels and TLR3 signals capable of increasing cytokines and chemokines in PTC cells* in vitro* are consistent with the existence of immune cell infiltrates* in vivo*, based on related studies suggesting that elevated TLR3/TLR3 signals in HT are associated with immune cell infiltrates [109].

Several authors have found* RET/PTC* rearrangements in nonneoplastic thyroid lesions, such as CLT [110–112]. Muzza et al. investigated clinical and molecular features of 128 patients with PTC and concurrent CLT and 215 patients with PTC alone. The two groups did not show significant differences in clinical and prognostic features, whereas they harboured a significantly different genetic background, with* RET/PTC1* being more represented in PTCs associated with autoimmunity and* BRAF(V600E)* in patients with PTC alone. A* RET/PTC* rearrangement was also found in 41% of nonneoplastic thyroid tissues, contralateral to tumors harbouring either* RET/PTC* or* BRAF* mutations. The strong association between* RET/PTC1* and thyroiditis points to a critical role of this oncoprotein in the modulation of the autoimmune response [113]. Rhoden et al. investigated samples of both CLT and PTC. They found that low-level* RET/PTC* recombination occurs in nonneoplastic follicular cells in CLT and in a subset of PTC, suggesting that overlapping molecular mechanisms may govern early stages of tumor development and inflammation in the thyroid [114]. Kang et al. investigated the* RET/PTC-RAS-BRAF* in oxyphil cells in the vicinity of large lymphoid HT infiltrates and in malignant PTC cells. The expression of RET, nuclear RAS, and ERK proteins is greatly enhanced in both PTC and HT oxyphil cells. Then, the RET/PTC-RAS-BRAF cascade may be associated with the development of PTC and oxyphil cell metaplasia in HT. These results show the possibility of a molecular link between oxyphil cell metaplasia in HT and the progression of PTC [115].

Different mechanisms could also explain the association between* CLT* and* RET/PTC* rearrangement. The first hypothesis is that inflammation might facilitate the rearrangement. It is worth noting that the production of free radicals, cytokine secretion, cell proliferation, and other phenomena correlated with inflammation might predispose to the rearrangement in follicular cells [116]. It is well known that leukocytes recruited under an inflammatory scenario physiologically secrete reactive oxygen species and reactive nitrogen species. However, these highly reactive molecules induce the production of peroxynitrite and other mutagenic agents, leading to DNA damage [117]. If tissue damage persists, these free-radicals secreted by immune cells may induce point-mutations, DNA rearrangements, and double-strand breaks [116, 118].

Guarino et al. proposed that cytokines and chemokines released by inflammatory tumor stroma could sustain the survival of thyroid cells in which* RET/PTC* rearrangements randomly occur, thereby allowing the selection of clones that acquire additional genetic lesions and become resistant to oncogene-induced apoptosis [116], which is the second hypothesis. In fact, some studies suggest that* RET/PTC* might induce apoptosis [119, 120]. Some studies suggest that thyroid cancer cells, like other epithelial cancer cells, may produce inflammatory factors that may facilitate cell survival, preventing apoptosis. These data reinforce this second hypothesis of interaction between* RET/PTC* rearrangement and thyroid inflammation. Stassi et al. demonstrated that thyroid cancer variants produced interleukin-4 and interleukin-10, which increased antiapoptotic molecule (Bcl-2 and Bcl-xL) levels and protected thyroid cells from chemotherapeutic agents [121]. Conticello et al. found that IL-4 protects tumor cells (primary prostate, breast, and bladder cancer) from CD95- and chemotherapy-induced apoptosis by the upregulation of antiapoptotic proteins, such as cFLIP/FLAME-1 and Bcl-x(L) [122]. Todaro et al. identified the fact that primary epithelial cancer cells from colon, breast, and lung carcinomas express high levels of the antiapoptotic proteins PED, cFLIP, Bcl-xL, and Bcl-2. These cancer cells produced interleukin-4 (IL-4), which amplified the expression levels of these antiapoptotic proteins and prevented cell death induced upon exposure to drug agents. Furthermore, exogenous IL-4 was able to upregulate the expression levels of these antiapoptotic proteins and potently stabilized the growth of normal epithelial cells, making them apoptosis-resistant, suggesting that IL-4 acts as an autocrine survival factor in epithelial cells [123].

The third hypothesis is that* RET/PTC* rearrangement might induce chronic inflammation. This hypothesis is fairly supported by literature, since many investigations suggested that* RET/PTC* would favor the proinflammatory microenvironment through production of different molecules by thyrocytes [124–127]. In fact, Russell et al. found that* RET/PTC3* alone increases nuclear NF-kappaB activity and secretion of MCP-1 and GM-CSF. In addition, transfer of RP3-expressing thyrocytes into mice* in vivo* attracted dense macrophage infiltrates, leading to rapid thyroid cell death [128]. The same group of authors found that IL1-alpha, IL1-beta, IL6, TNF-alpha, and the Cox2 enzyme are produced by RET/PTC3-transgenic thyroid tissue, but absent from nontransgenic thyroids, supporting the notion that oncogene-induced cytokine secretion is important for the development and progression of thyroid carcinomas in genetically permissive hosts [129]. Genes coding for proteins involved in the immune response (prostaglandin E2, microsomal prostaglandin E2, cyclooxygenase 2, and IL24) have been suggested to be induced by RET/PTC, indicating that the expression of the oncogenic fusion protein RET/PTC is critical not only in thyroid cancer pathogenesis but also in the elicitation of inflammatory response [130–132].

*BR* *AF* ^*V*600*E*^ mutation is a common genetic alteration in PTC. Kim et al. found that, in Korean patients with PTC, *BRAF* ^*V*600*E*^ mutation is associated with a lower frequency of background HT [133]. Likewise, Muzza et al. found that *BRAF* ^*V*600*E*^ is more represented in PTC without concurrent autoimmunity [113]. In fact, studies on melanoma cells have given clues about mechanisms linking* BRAF* mutation and immune response; and IL-10, VEGF, IL-6, and IL-8 are thought to be induced by *BRAF* ^*V*600*E*^ mutation [134, 135].

The genomic alterations in BRAF-MAPK signaling pathway have been recently demonstrated to be associated with immune escape mechanisms. Smallridge et al. performed a RNA sequencing in order to identify genes differentially expressed between* BRAF*-mutant and* BRAF* wild-type tumors and to correlate changes to patient clinical status. Immune/inflammatory pathways were expressed at a lower level in* BRAF*-mutant tumors due to either suppression of immune/inflammation genes in BRAF-mutant tumors or increased expression by the tumor or by infiltrating lymphocytes in* BRAF* wild-type tumors [136]. Interestingly, the authors observed that major histocompatibility complex, class I, G (HLA-G) gene, a nonclassical HLA class 1 molecule, was overexpressed in the* BRAF*-mutant tumors [136]. HLA-G evokes several immunosuppressive functions, including inhibition of cytotoxic CD8+ T cells and natural killer cells, altering dendritic cell function, shifting from Th1 to Th2 T cells, and favoring the promotion of immune escape. They suggested that upregulation of HLA-G in PTC epithelial cells would be directly responsible for impaired immune surveillance in* BRAF*-mutant compared with* BRAF* wild-type tumors [136].

**Figure 2:**
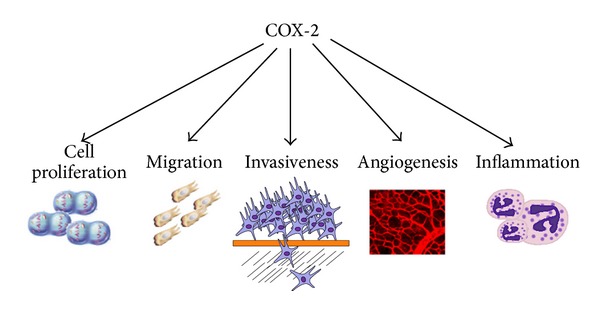
COX-2 is a catalyzing enzyme that converts cellular arachidonic acid to prostaglandin, contributing to trigger inflammation. Many reports have demonstrated that COX-2 is not only responsible for cell proliferation and transformation but also responsible for inducing angiogenesis. In addition, migration and invasiveness have been associated to overexpression of COX-2.

Special attention should be given to cyclooxygenase-2. Cyclooxygenases (COX) components of the arachidonic acid cascade are a family of catalyzing enzymes that convert cellular arachidonic acid to prostaglandin. There are two major cyclooxygenases, COX-1 and COX-2 [137]; COX-2 is an inducible gene [138]. Recently, it has been reported that COX-2 is overexpressed in many human tumors [139–142]. This result has suggested that COX-2 may play an important role in the carcinogenesis and progression of tumors (Figure 2). Some reports have observed that COX-2 is not only responsible for cell proliferation and transformation but also for inducing angiogenesis [143, 144]. In a chemical-induced thyroid carcinogenesis model, Ota et al. observed that COX-2 might play important roles in follicular cell proliferation but do not affect tumor induction [145].

Although some reports have suggested that COX-2 expression is not different among thyroid lesions [146], Ji et al. observed that COX-2 expressions in undifferentiated carcinomas and medullary carcinomas were higher than in PTC and FTC. In addition, the expression of COX-2 in thyroid adenomas was higher than that in normal tissues. These findings suggest that overexpression of COX-2 may be an early event in the progression of thyroid neoplasms [147]. A close relationship between COX-2 and VEGF expression was also noted [147]. Likewise, Siironen et al. observed that VEGF-C expression correlated strongly with COX-2 expression [148]. Cornetta et al. analyzed COX-2 expression in twenty paraffin-embedded human tissue specimens, including normal, inflammatory, and neoplastic thyroid sections. Immunohistochemical staining confirmed the presence of COX-2 in thyroid epithelial neoplasms, including PTC and FTC. Moreover, COX-2 expression was observed in patients with Hashimoto's thyroiditis but not observed in normal thyroid tissue, multinodular goiter, or anaplastic carcinoma, suggesting that COX-2 expression in both of these thyroid pathologies may provide a basis for the relationship between carcinogenesis and autoimmunity [149].

Ito et al. studied COX-2 expression in both differentiated and undifferentiated thyroid cancers. They observed that COX-2 expression was reduced in cases with old age, large size, advanced stage, satellite tumors, and the presence of solid, scirrhous, or trabecular growth patterns. Undifferentiated carcinomas less frequently overexpressed COX-2 [150]. Lim et al. observed an association between the absence of COX-2 expression and multiplicity and bilaterality in papillary thyroid microcarcinomas [151].

Kajita et al. investigated COX-2 expression in PTCs and matching normal tissues. COX-2 protein, but not mRNA expression, was greater in PTC when compared with normal thyroid tissues. From* in vitro* study, they concluded that COX-2 also has a role in PTC growth, since a specific inhibitor of COX-2 regulates PTC cell proliferation [152]. Conversely, Scarpino et al. investigated the biological role of COX-2 in PTC cells by treating PTC cell lines with the specific COX-2 inhibitor NS-398. It was found that COX-2 inhibitor treatment significantly reduced the migration and invasiveness of tumor cells but did not alter cell proliferation [153].

In contrast to DTC, anaplastic thyroid carcinoma is one of the most aggressive neoplasms in humans and it accounts for 5–15% of primary malignant thyroid tumors [154]. Since anaplastic thyroid carcinoma is a rare cancer, the small number of available cases has impaired the opportunity to better understand tumor biology and the natural history of this disease. In addition, the factors that may affect the response to treatment and survival are not well known. There are tiny evidences that immune system may be evoked in ATC. Significant amounts of colony-stimulating factor and IL-1 alpha were detected in the conditioned media of undifferentiated giant cell carcinoma of the thyroid [155], as suggested by case report [156]. Sato et al. reported two cases of anaplastic thyroid carcinoma associated with neutrophilia and elevated IL-6 and macrophage colony-stimulating factor [157]. Immunohistochemical staining revealed that carcinoma cells themselves produce IL-6 regardless of the types of carcinoma cells [157]. Suzuki et al. obtained peripheral blood mononuclear cells from 49 patients with thyroid cancer, 18 patients with noncancerous thyroid diseases, and 22 healthy volunteers. The MDSC levels were found to be higher in patients with any type of thyroid cancer, patients with anaplastic thyroid carcinoma, and patients with medullary thyroid carcinoma when compared to patients with noncancerous thyroid diseases, suggesting that MDSCs could be new targets for immunotherapy against anaplastic thyroid carcinoma [158]. However, further studies are required to verify this hypothesis.

## 6. Immune Cell Infiltration and DTC

**Figure 3:**
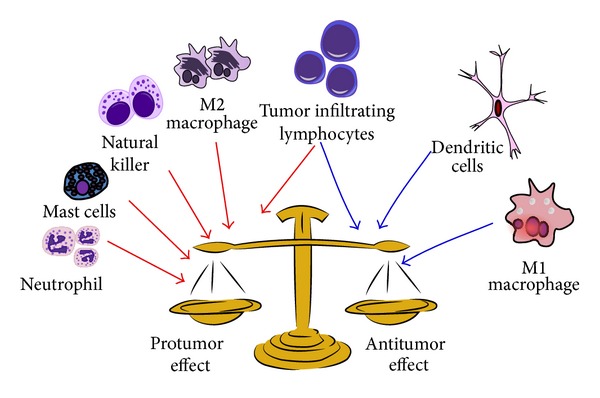
Mixture of immune cells may infiltrate thyroid cancer microenvironment. Some cells (red arrow) may activate different protumor mechanisms that enhance tumor progression. On the other hand (blue arrow), some cells may lead to antitumor immune response that is associated with favorable prognosis.

Innate immune response is a branch of the immune system that protects the host in a nonspecific manner. Innate immunity is the first line of defensive response, and effective mechanisms engaged in innate immune response are natural barriers (e.g., skin, gastric mucosa, and respiratory mucosa) and some immune cells (e.g., neutrophils, eosinophils, and mast cells). In fact, major effector cells of the immune system that directly target cancer cells include natural killer cells (NK), dendritic cells (DC), macrophages, polymorphonuclear leukocytes (including neutrophils, eosinophils, and basophils), mast cells, and cytotoxic T lymphocytes. NK cells, DC, polymorphonuclear cells, mast cells, and macrophages are first-line effectors to damaged cells and cancer cells (Figure 3) [159].

The importance of the innate immune system in limiting cancer progression has been highlighted recently with the following direct molecular interactions between cancers and innate immune effector cells [159]. Probably tumors are able to sense the innate immune system. Innate immunity is thought to produce signals that drive antigen presentation toward induction of adaptive immune response. The field of innate immune sensing is growing rapidly, with several distinct families of proteins having been identified and additional family members still uncharacterized [160].

Apetoh et al. described, in both mice and humans, that the activation of tumor antigen-specific T-cell immunity involves secretion of the high-mobility-group box 1 alarmin protein by dying tumor cells and the action of high-mobility-group box 1 on toll-like receptor 4 (TLR4) expressed by DCs. During chemotherapy or radiotherapy, DCs require signaling through TLR4 and its adaptor MyD88 for efficient processing and cross-presentation of antigen from dying tumor cells [161]. Then, TLR4 would be an important key for antitumor effective immune response.

Toll-like receptors belong to a family of receptors that take part in innate and adaptive immunity by activating both T- and B-cell-mediated immune responses [4]. A minimum of 11 human TLRs are activated by various bacterial and viral components and also by endogenous factors [162–164]. The activation of TLR-2 and TLR-4, the principal receptors for bacterial antigens (bacterial lipopolysaccharides), leads to secretion of cytokines, chemokines, and other proinflammatory mediators [4]. Hagström et al. studied the immunohistochemical expression of TLR-2 and TLR-4 in 127 follicular thyroid neoplasms, both in adenomas and in carcinomas including oxyphilic tumors. In follicular thyroid neoplasias, TLR-2 and TLR-4 expression was predominantly cytoplasmic. Patients for which tumors were negative for TLR-4 presented with 5-year survival of 67%, whereas patients for which tumors were positive for TLR-4 presented with 5-year survival of 92%. TLR-2 expression showed no correlation with any clinical or histological parameters in follicular thyroid carcinoma and follicular thyroid adenoma [4]. TLR-2 and TLR-4 mRNA and protein expression have been reported in thyroid cells* in vitro* [165, 166], and these TLRs promote tumor progression in cancer by activating cell proliferation and taking part in tumor invasion [162, 167]. Lack of expression, however, may imply immune cell recruitment failure and may lead to invasion of unattended tumor cells [4].

Human leukocyte antigen (HLA, also known as major histocompatibility complex class I) plays a pivotal role in immune tolerance and a paradoxical part in cancer. Avoiding HLA expression could prevent tumor cells destruction, acting in favor of immune evasion. Dardano et al., studying 183 PTC patients, observed that those with detectable plasma soluble HLA-G levels showed a higher aggressive behavior than those without [168]. HLA expressed by tumor cells was, according to Nunes et al., significantly associated with an increased occurrence of lymph node metastasis and capsular invasion [169]. These results suggest that abnormal HLA expression may be a tumor mechanism used to impair antitumor immune response and HLA may be highlighted as a potential marker of aggressiveness and poor prognosis.

### 6.1. Natural Killer Cells

Natural killer cells are important effector cells of innate immunity. They are able to recognize and destroy pathogens [170]. Phenotypically, NK cells are defined by the expression of CD16 and CD56 surface markers, which form a lymphocyte subpopulation without specific markers for B- or T-cells, and NK cells lack the TCR/CD3 complex [171]. Functionally, NK cells are able to produce several cytokines, such as tumor necrosis factor-*α*, interferon-*γ*, and granulocyte-macrophage colony-stimulating factor (GM-CSF) [172]. The emerging notion is that NK cells are not only cytolytic effector cells against microbe-infected cells or tumor cells. Rather, NK cell-mediated cytotoxicity and cytokine production impact DC, macrophages, and neutrophils and endow NK cells with regulatory function, affecting subsequent antigen-specific T- and B-cell responses [172]. Gogali et al. investigated infiltration of NK cells in thyroid glands of 65 patients with PTC and 25 with thyroid nodular goiter. PTCs were enriched with NK cells and authors found an increased number of NK cells in PTC tissue when compared with thyroid nodular goiter tissue. In addition, they observed an inverse correlation between NK cell infiltration and tumor stage, with decreased NK cell infiltration in advanced stages [173]. This result suggests the weakness of the innate immune response towards cancer spread in advanced stages.

NK cells can be divided into two functional subsets based on their surface expression of CD56. CD56dim are cytotoxic cells, whereas CD56bright play an immunoregulatory role [174]. These NK cell subsets present differences in their cytotoxic potential, cytokine production, and response to cytokine activation [175, 176]. Liapi studied the distribution of cytotoxic and immunoregulatory NK subpopulations in tissue and blood samples from patients with PTC and nodular goiter. The distribution of immunoregulatory and cytotoxic NK cell subpopulations in the peripheral blood was similar in patients with PTC, nodular goiter, or healthy donors. PTC microenvironment presented increased immunoregulatory NK cells. Comparison of NK cell subpopulations between PTC and goiter revealed that cytotoxic cell numbers were significantly higher in goiter than in PTC. In contrast, immunoregulatory cells showed a higher infiltration in PTC than goiter tissues, pointing to the fact that an immunoregulatory pattern of NK cells is required for thyroid carcinogenesis [174]. Interestingly, cytotoxic NK cell tissue infiltration positively correlated with advanced stages of PTC. In contrast, the immunoregulatory NK cell population was negatively associated with tumor stage in patients with PTC. The tumor microenvironment can be the site where immature immunoregulatory cells may transform into mature cytotoxic NK cells, but this developmental program is not entirely fixed, and mature NK cells can be reeducated by local, environmental factors. This could probably explain the gradual increase or decrease in the percentages of NK cell subpopulations correlating with the tumor stage in their study [174].

### 6.2. Dendritic Cells

Dendritic cells represent a small subset of immune cells derived from bone marrow and found in nearly every tissue in the human body [130]. DCs are considered to be professional antigen-presenting cells based on their ability to present antigen in the context of MHC class II and costimulatory molecules. They are, therefore, extremely efficient stimulators of immunity and are thought to be key players in initiating the body's immune response [131]. The potential for DCs to amplify immune function in an antigen-specific manner makes them ideal candidates for cancer immunotherapy, which attempts to eradicate tumors by manipulating the body's own innate immune mechanisms [131, 132].

Tsuge et al. described the infiltration of DC in DTC. Thyroid tissues were obtained at thyroidectomy from 85 patients with primary thyroid cancer, most with PTC. PTC had a higher frequency of CD1a+ immature DCs than other thyroid tumors. DCs positive for chemokine receptor-6 (CCR-6) were densely accumulated in PTC [177]. A series of 527 consecutive cases of thyroid carcinoma treated by total thyroidectomy was investigated by Ugolini et al. They applied histopathological and immunohistochemical methods to investigate inflammatory infiltrate in these tumors. Immature DCs were detected in PTCs and markedly reduced in poorly differentiated and undifferentiated thyroid carcinomas, suggesting the protective role of DC and infiltrating lymphocytes against thyroid tumors [178]. In fact, Hilly investigated specimens from 69 consecutive patients with PTC, counting S100+ DC. DC density in PTC correlated with the thyroiditis grade and DC density in surrounding areas of thyroiditis [179]. This association suggests that intratumoral infiltration of DC may be linked to a more complex immunological phenomenon caused by carcinogenesis.

Pusztaszeri et al. quantitatively assessed the presence of DCs that were positive for CD1a in cytologic samples of histologically confirmed PTC and in a control group of benign thyroid nodules. CD1a-positive DCs were identified in 97% PTCs in thyroid fine-needle aspiration specimens. DCs were largely present in two distinct patterns: either as isolated DCs in the background or as associated with tumor cells. Both thyrocyte-associated DCs and background DCs were more numerous in PTC fine-needle aspirations than in benign thyroid nodule fine-needle aspirations, but only the thyrocyte-associated group of DCs was statistically significant, suggesting that malignant cells are able to recruit DCs during carcinogenesis [180]. Likewise, Proietti et al. selected 91 consecutive cases of follicular variant of PTC, in which we evaluated the presence of mature and immature DCs. In intratumoral and peritumoral areas, the expression of immature marker of DC was significantly higher in follicular variant of PTC than in adenomas. Expression of DC immature marker was comparable in the extratumoral compartment [181].

Scarpino et al. observed that most DCs are located in correspondence with the advancing edge of the tumor. Established primary cultures of neoplastic thyroid cells and normal cells (from the tumor-free contralateral lobe of the same patients) obtained from eight thyroids were removed surgically. Supernatants of both normal thyroid cells and tumors cells are able to produce chemokines that recruit DC [182]. This result is an apparent contradiction, since DCs are rare in normal thyroid tissue, and raises the possibility that culture conditions may cause stimulatory signals for normal thyroid cells. In fact, authors previously demonstrated that normal thyroid cells of primary cultures, but not normal thyroid cells of tissue sections, express high levels of Met protein and urokinase-type plasminogen activator receptor, and both factors stimulate DC recruitment. In addition, Met protein is expressed in Hürthle cells of Hashimoto thyroiditis and hyperfunctioning thyrocytes of Graves' disease [182–185], indicating that upregulation of Met receptor is an early event in thyroid cell alteration. This result may explain the rare infiltration of DC in benign thyroid lesions [180].

### 6.3. Mast Cells

Mast cells play an important role in inflammatory processes, particularly in the initiation of the immune response [186]. They are residents of normal connective tissue and have been widely discussed for their role as tumor promoters in tumor pathogenesis [187, 188]. To assess the role of mast cells in human thyroid cancer, Melillo et al. compared the density of tryptase-positive mast cells in 96 PTCs versus normal thyroid tissue from 14 healthy individuals. Mast cell density was higher in 95% of PTCs than in control tissue. Mast cell infiltrate correlated with extrathyroidal extension of PTCs. Authors show that thyroid cancer cell-line-derived soluble factors induce mast cell activation and chemoattraction* in vitro*, suggesting that mast cells are recruited to tumor microenvironment during malignant transformation. In addition, Melillo et al. found that mast cells induced thyroid cancer cell invasive ability, survival, and DNA synthesis* in vitro*, suggesting that mast cells have a pivotal role in thyroid tumor progression and dissemination [189]. Proietti et al. evaluated the presence and distribution of mast cells in follicular variant of PTC and follicular adenoma. Interestingly, they were significantly highly expressed in the peritumoral compartment of follicular variant of PTC. Considering only the follicular variant of PTC group, there was also a significant correlation between the abundance of mast cells and the infiltrative pattern of the tumor [181]. These data corroborate the results of Acikalin et al., who suggested a protumoral role for mast cells based on the tight association between these cells and the promotion of microangiogenesis in tumor tissues [190].

### 6.4. Macrophages

Cancer cell recruits monocytes from circulation. Herein, monocytes are induced to differentiate into macrophages. Phenotypically, macrophage can be recognized by the expression of CD68. Functionally, there are two different subsets of macrophages: M1 and M2. M1 main function is phagocytosis in response to bacterial stimuli and/or Th1 cytokines, while the main function of M2 is immunosuppression and trophic activity in response to Th2 cytokines [191, 192].

Herrmann et al. observed that 75 thyroid carcinomas of follicular cell origin presented rising levels of CD68 positive cell infiltration associated with dedifferentiation. Positive correlations could be demonstrated between the density of CD68-, CD3-, and CD45RO-positive cells and between the density of CD68-, and CD3-, and CD45RO-positive cells and vascularisation. These correlations were expected, as the interaction of CD68-positive cells and T-lymphocytes results in the production of angiogenic factors, ultimately leading to better vascularisation of the tumor [193].

Ryder et al. characterized the density of tumor-associated macrophages (TAM) in well-differentiated, poorly differentiated, and anaplastic thyroid carcinoma and correlated TAM density with clinicopathological parameters. In total, 27% of well-differentiated thyroid carcinoma, 54% of poorly differentiated thyroid carcinoma, and 95% of anaplastic thyroid carcinoma had an increased density of CD68(+) TAMs. Increased TAMs in poorly differentiated thyroid carcinoma were associated with capsular invasion and extrathyroidal extension, and decreased cancer-related survival was compared with poorly differentiated thyroid carcinoma with a low density of TAMs [194]. The same group used BRAF-induced PTC mouse models to examine the role of TAMs in PTC progression. Conditional activation of BRAF in murine thyroids was associated with a significant increase in the major TAM chemoattractants. They conditionally depleted CCR2-dependent TAMs during BRAF induction, resulting in smaller tumors and decreased proliferation. Phenotypic analysis demonstrated an increased expression of M2-related genes such as Ccr2, arginase1, Ccl22, and IL-10, whereas M1-specific markers (IL-12 and ROS) were not increased. Selectively depleting TAMs, during advanced stages of PTC, induce tumor regression. Authors suggested that, as TAMs promote PTC progression, these cells may be a rational therapeutic target for patients with refractory advanced PTCs, particularly poorly differentiated and anaplastic thyroid carcinoma [195]. However, probable macrophages do not exert the same role in poorly differentiated thyroid carcinoma and well-differentiated thyroid carcinoma.

We studied 398 patients with DTC and 132 with nonmalignant tissues [196]. We found TAM more frequently in more aggressive cases, with metastasis at diagnosis; however, paradoxically, macrophage infiltration correlated with improved disease-free survival [196]. How can we explain the apparent contradictory results? It is possible that these different results lie on different microenvironments. In fact, Fiumara et al. studied 121 well-differentiated PTCs and found tumors with TAM and* in situ* evidence of active neoplastic cell phagocytosis [197]. Neoplastic cell phagocytosis by macrophages was positively correlated with infiltration of lymphocytes and DCs, while it was negatively correlated with vascular invasion [197]. They also found a trend of a reduced risk of distant metastases at follow-up in cases with TAM that, in addition, was associated with lymphocytic infiltration [196, 197]. Again, an infiltration of mixture of immune cells can be noted in thyroid tumor microenvironment, and the prognosis of patients with DTC may be influenced by the complex interaction between these immune cells.

### 6.5. Tumor Infiltrating Lymphocytes

Unfortunately, macrophages and other innate immune cells cannot always efficiently eliminate tumor cells. However, macrophages and DCs play an important role in the initiation and subsequent direction of adaptive immune responses mediated by lymphocytes [198]. In addition to providing a more versatile defense response, lymphocytes provide increased protection against subsequent reinfection with the same pathogen [198]. Then, unlike innate immune responses, adaptive responses are highly specific to the particular antigen that induced them. They can also provide long-lasting protection through a mechanism called memory. A person who recovers from measles, for example, is protected for life against measles by the adaptive immune system, although not against other common viruses, such as those that cause mumps or chickenpox [199]. How does the adaptive immune system work in a context of tumor development and progression?

Matsubayashi et al. were concerned about the influence of tumor infiltrating lymphocytes (TIL) and the prognosis of patients with PTC. They studied 95 patients with PTC who received primary surgical treatment in 1983 and were followed until 1992. They observed that 36 patients (group A) with PTC also presented with an associated TIL, whereas 59 patients (group B) had no TIL. Recurrence of the tumor was found in only one patient in group A (2.8%), but in 11 patients in group B (18.6%). The percentage of patients free from recurrence over a 10-year follow-up period in group A was significantly higher than that in group B, suggesting that TIL might be used for predicting a favorable prognosis [1]. Villagelin et al. investigated 157 consecutive patients with PTC. They were classified by the degree of TIL: diffuse, peritumoral (only in or around the tumor), or absent TIL. After a mean follow-up period of 8 years, they observed a significantly high recurrence in the absent lymphocyte infiltration group when compared with patients from the diffuse and peritumoral lymphocyte infiltration groups, suggesting that the presence of TIL may favor the prognosis of patients with PTC [200]. However, lymphocytes are pool of cells in which multiple phenotypes can be found. Then, it is very important to assess different subsets of TIL to study prognostic prediction.

Modi et al. examined 21 PTCs from patients aged 21 years or younger for the presence of CD4+ (helper), CD8+ (killer), CD19+ (B cells), and CD56+ (natural killer) cells. During follow-up, none of the PTCs containing either CD8+ lymphocytes or the combination of CD4+, CD8+, and CD19+ lymphocytes recurred. Unfortunately, the cohort was too small and the follow-up inadequate to provide accurate information on the clinical impact of these immunological findings [2]. French et al. obtained opposite results. They investigated whether TIL, in the absence of concurrent CLT, contributes to disease severity. One hundred PTC patients were analyzed for concurrent CLT and TIL, and 10 PTC patients with TIL were assessed for lymphocyte subsets by immunohistofluorescence. Patients with TIL exhibited higher disease stage and increased incidence of invasion and lymph node metastasis compared with those without TIL or with concurrent CLT. CD4(+) T-cell frequency correlated with tumor size, FoxP3(+) regulatory T-cell (Treg) frequency correlated with lymph node metastasis, and CD8 to Treg ratio inversely correlated with tumor size [5]. What does the study of TIL subsets say to us?

CD4+ T cells play a central role in orchestration of the immune response. Naive CD4+ T-cells may differentiate into one of at least four functionally distinct populations of cells: Th1, Th2, Tregs, or Th17. Th1 polarization is characterized by the production of interferon (IFN)-*γ* and supports the cytotoxic response mediated by CD8^+^ T. In fact, IFN-*γ* has direct effects on tumor cell immunogenicity and thus plays an important role in promoting tumor cell recognition and elimination [87]. Conversely, Th2 polarization stimulates humoral immunity, and recent advances support the hypothesis that enhanced states of local humoral and innate immune activation, in combination with suppressed cellular immunity and failed cytotoxic T-cell antitumor immunity, alter cancer risk toward tumor promotion and progression. In general, Treg are identified as FoxP3+ lymphocytes [88]. According to the origin, Treg can be categorized in natural Treg (originate in the thymus) or inducible Treg (originate under specific conditions) [86]. Both are thought to contribute to tumor-specific T-cell tolerance [201]. The recent discovery of Th17 cells and their important role in host protection against infectious pathogens and in the pathogenesis of various inflammatory and autoimmune diseases have resulted in an explosion of immunological research. However, their part in human cancer is still under investigation [202]. Recently, Moretti et al. evaluated indoleamine 2,3-dioxygenase 1 (IDO1) expression in thyroid carcinoma. IDO1 catalyzes tryptophan degradation to kynurenine and appears to exert an immunosuppressive function as part of an acquired mechanism of immune escape mediated by the inhibition of lymphocyte proliferation and survival and by the induction of Treg. Authors demonstrated that IDO1 expression was elevated in thyroid carcinoma compared to normal thyroid tissue. In addition, IDO1 expression magnitude was correlated with both infiltration of Treg and gain of aggressiveness (PTCs and medullary thyroid carcinomas ≪ anaplastic thyroid carcinomas) [203].

Aiming at better characterizing TIL in DTC microenvironment, we studied 398 patients, 253 with PTC, 13 with follicular thyroid cancers, and 132 with benign thyroid lesions. Our data demonstrated that the presence of concurrent CLT and infiltration of CD4+, CD8+, CD20+, Th17, and Treg cells are associated with favorable prognostic features in patients with DTC. Immune cells were found to infiltrate malignant tissues more often than benign lesions, suggesting an immune reaction of the organism against transformed cells [196]. However, how the immune response may alter the outcome of patients with DTC is not that simple. Prognosis seems to be an output of complex interactions between the immune system and tumor cells. In fact, our data indicate that immune cell infiltration is closely associated with the immunohistochemical profile of the DTC specimens examined, including proteins whose expression might indicate tumor differentiation and progression, demonstrating tumor antigenicity (NIS, MUC1, PTEN, ATM, and B7H1) [204–207]. A special attention must be given to B7H1.

Tumors may escape from immune response through several mechanisms. One of these mechanisms is the upregulation of B7H1 expression. B7H1 is a cell surface glycoprotein and aberrant tumor expression of B7H1 is thought to be associated with inhibition of the immune system [208]. As expected, B7H1 expression in tumor cells has been associated with poor prognosis in some epithelial cancers [209]. We demonstrated that both B7H1 protein and B7H1 mRNA are upregulated in differentiated thyroid carcinomas, contrasting with the low levels displayed by benign tissues [210]. We also observed an association between high B7H1 mRNA levels and an aggressive phenotype, like higher stages at presentation and increased age at diagnosis, suggesting that high levels of B7H1 expression may identify individuals who need a more aggressive approach [210]. Interestingly, we observed that lymph node metastatic tissues, compared with respective matched primary tumor tissue, had lower levels of B7H1, indicating T-cell exhaustion [210]. In fact, French et al. recently described high levels of IFN+/CD8+ T-cells in metastatic lymph node tissues excised during the initial surgery, at patient presentation [211]. They found that proliferating lymphocytes were evident in tumor-involved lymph node metastases that were enriched with B7H1-ligand+ lymphocytes [211]. Authors hypothesized that the presence of metastases may promote an IFN+ response, suggesting the generation of an antitumor response, which may impair tumor evasion. In fact, this could explain the decrease in B7H1 expression that we observed in our matched metastases, indicating that T-cell exhaustion is a mechanism of tumor progression.

Gupta et al. hypothesized that TIL with a high proliferation index would be found in thyroid cancers from children and young adults and would be associated with improved disease-free survival. Using immunohistochemistry, they examined 39 childhood PTC, 9 FTC, 2 medullary thyroid carcinomas, 11 benign thyroid lesions, and 2 normal thyroid glands for the presence of lymphocytes and lymphocyte proliferation. Disease-free survival did not correlate with the presence or number of lymphocytes per high-power field. In contrast, disease-free survival was significantly improved for thyroid cancers with the greatest number of proliferating lymphocytes per high-power field [6].

## 7. Medullary Thyroid Carcinoma and Immune Response

Medullary thyroid carcinoma (MTC), arising from parafollicular, calcitonin-producing C-cells, represents an aggressive, usually slow-growing tumor occurring in both sporadic and familial forms such as multiple endocrine neoplasia type 2 [212].

Sporadic MTC harbors* RET* gene somatic mutations in up to 50% of cases, and* RAS* family gene mutations occur in about 10% [213]. Simbolo et al. investigated 20 surgically resected sporadic MTCs. Thirteen (65%) MTCs harbored a* RET* mutation; four cases harbored a* RAS* mutation: three in* HRAS* and one in* KRAS* [213]. On the other hand, hereditary MTC is one of the most successful applications of genetics knowledge in medical practice. The discovery of mutations in the* RET* protooncogene resulting in variable onset and severity of multiple endocrine neoplasia type 2 was the first step in developing direct genetic testing for at-risk individuals. Patients with germline* RET* mutations may undergo risk assessment and appropriate intervention based on specific mutations [214]. Clinicians are therefore able to make better therapeutic choices guided by an informative biomarker, reinforcing the current concept of an increasingly personalized medicine [214].

No conventional therapy has been established for the metastatic state, since MTC usually presents low chemosensitivity and radiosensitivity. In contrast, C-cells of the thyroid gland are of neuroendocrine origin and may therefore be highly susceptible to an immune attack, as are other neuroendocrine cells such as *β*-cells of the pancreas in autoimmune type 1 diabetes mellitus or parathyroid cells in autoimmune hypoparathyroidism [215–217]. All these features encouraged immunologists and endocrinologists to test cancer immunotherapy against MTC.

It has been known for a long time that the immune system plays a role in MTC [218, 219]. In fact, Müller et al. [220] observed an increase of FoxP3+ lymphocytes in peripheral blood of patients with MTC but not in patients with benign goiter; this increase also correlates with findings in lymph nodes and thyroid gland. Authors found that the number of FoxP3+ cells correlated with the patients' prognosis. After clinical staging (International Union against Cancer-UICC-stages) of MTC patients, triplication of FoxP3+ lymphocytes could be observed from MTC < UICC II to MTC > UICC II, suggesting that the immune system could interfere in outcome of patients with MTC.

Different proteins are known to be the possible target molecules for immunotherapy in MTC. Calcitonin is the most important target molecule. Zhang et al. performed an epitope mapping with monoclonal antibodies and the highest immunogenicity of the central region of calcitonin (amino acids 13–21) was shown [221]. Haupt et al. [222] investigated how immunogenic calcitonin is. Antigen-encoding expression plasmids were delivered intradermally by gene gun. One group of mice received DNA encoding human precursor protein preprocalcitonin only. Two groups were coinjected with mouse cytokine genes. They observed in lymphocyte proliferative assays substantial proliferation against human precursor protein preprocalcitonin in mice coinjected with the granulocyte-macrophage colony-stimulating factor gene, in contrast to mice vaccinated with human precursor protein preprocalcitonin expression plasmid only, suggesting that cellular and humoral immune responses against human precursor protein preprocalcitonin can be generated by DNA immunization. Conversely, codelivery of IFN-*γ* expression plasmid resulted in a decreased antibody response against human precursor protein preprocalcitonin, probably due to enhanced Th1-like immunity. Wuttke et al. [223] studied amino acid-modified calcitonin. Mice were immunized over six months with monthly injections of amino acid-modified CT-pulsed dendritic cells. CT-immunized mice showed evidences of antitumor immune response, with an intratumor infiltration with CD8+ T lymphocytes. Importantly, they also found a diminished tumor outgrowth and a decrease in serum CT levels compared with control mice, suggesting that amino acid-modified CT is recognized from the immune system leading to an effective specific antitumor immune response. This result raises the idea that immunotherapy strategies against MTC would be key point for MTC treatment.

Anti-MTC cancer vaccines are the most important strategy of immunotherapy against MTC. Therapeutic cancer vaccines are intended to activate the immune system for treating an existing tumor or preventing its recurrence. Since DCs are highly potent antigen-presenting cells and are able to orchestrate immune response, they represent an excellent tool for immunization against cancer [224]. Schott et al. reported on a clinical trial of DC vaccination in metastasized MTC [225]. Seven patients were enrolled in this study. Mature DCs were generated from peripheral blood monocytes in the presence of granulocyte-macrophage colony-stimulating factor, IL-4, and TNF*α*. After, DCs were loaded with calcitonin and carcinoembryonic antigen (CEA) peptide and repeatedly delivered by sc injections. In three of seven patients, clinical responses with a decrease in serum calcitonin and CEA were initially documented. One of these patients had a complete regression of detectable liver metastases and a significant reduction of pulmonary lesions. This patient presented with a calcitonin- and CEA-specific immunreactivity [225]. All seven patients developed a strong delayed-type hypersensitivity skin reaction, which was confirmed to be mediated by infiltrating CD4+ T-helper cells and CD8+ cytotoxic T-cells. Measurement of cytokine release from T lymphocytes demonstrated high posttreatment interferon-gamma secretion after stimulation with calcitonin in five patients. In contrast, antigen-specific interleukin-4 (IL-4) production was only slightly increased in four patients, suggesting that an induction of a Th1-dominated cellular immune response against calcitonin and CEA in the majority of patients does occur [226].

Stift et al. [227] evaluated the effectiveness of DC vaccines in 10 MTC patients. They generated autologous tumor lysate-pulsed DCs from all patients suffering from advanced MTC for repeated vaccination. Mature DCs were derived from peripheral blood monocytes, culture in the presence of granulocyte-macrophage colony-stimulating factor, interleukin 4, and tumor necrosis factor alpha with or without addition of IFN-gamma, loaded with tumor lysate, and finally further injected into a groin lymph node. Vaccination was well tolerated and induced a positive immunological response in all of the tested patients. Three patients had a partial response, one patient presented a minor response, and two patients showed stable disease at least 29 months after the start of immunotherapy. The remaining four patients had progressive disease [227].

Bachleitner-Hofmann et al. [228] performed a pilot trial of autologous DCs pulsed with tumor cell lysate derived from allogeneic MTC cell lines in 10 patients with metastatic MTC. DCs were injected into a groin lymph node at 3-week intervals, followed by serial calcitonin tumor marker measurements; radiological imaging and immunological* in vitro* tests were monitored. After a median follow-up of 11 months, three patients had stable disease, while seven progressed during treatment. In two patients with stable disease, calcitonin decreased below treatment levels, paralleled by a T-cell mediated immune response. Notably, treatment with DCs pulsed with a combination of different tumor cell lysates was followed by a calcitonin decrease in four patients who had previously experienced a calcitonin increase during monotherapy with DCs pulsed with a single lysate.

## 8. Conclusion

The first study that investigated immunotherapy in patients with follicular cell-derived thyroid cancer came out in 1975 [229]. Active immunotherapy was applied to three patients with thyroid cancer. Two patients, who were in the terminal stage of illness, could not develop generalized cell-mediated immunity and immunization did not alter the patients' rapid downhill course. One patient developed* in vitro* evidence of cell-mediated immunity against cancer tissue antigens, associated with decrease in tumor size. Unfortunately, four months after immunization, cell-mediated immunity was impaired in autologous plasma culture, but not in cultures in allogeneic normal plasma.

More than 40 years later, no significant advance has been made in immunotherapy for follicular cell-derived thyroid tumors. In part, this can be explained by the success of current therapy, which is able to solve the problem of 95% of patients diagnosed with DTC. However, it is noteworthy that immunotherapy is not the unique application of antitumor immune response. Infiltration of immune cells may predict outcome, and this is an important practical advance in the thyroid field, perhaps even more important than immunotherapy. The immune system gives us tools to identify more aggressive tumors and patients who may benefit from a more invasive approach, sparing the vast majority of patients with an indolent disease from unnecessary procedures. The recognition of immune aspects underlying thyroid cancer development and progression may help physicians to perform a more personalized approach to their patients. In fact, tumor immunology understanding has widened our boundaries.
